# Myositis due to COVID-19

**DOI:** 10.1136/postgradmedj-2021-139725

**Published:** 2021-02-15

**Authors:** Sadettin Uslu

**Affiliations:** Rheumatology, Ömer Halisdemir University Bor Physical Medicine and Rehabilitation Training and Research Hospital, Nigde 51200, Turkey

A 38-year-old man presented with dyspnoea and myalgia. Examination revealed tachycardia at 115 beats per minute and oxygen saturation (SpO_2_) of 88% on room air. He had lower extremity muscle weakness. Motor testing revealed a bilateral ankle plantarflexion deficit graded at 3/5 on the Medical Research Council muscle scale. All nerve reflexes were normal. The patient’s medical history was unremarkable.

**Figure 1:**
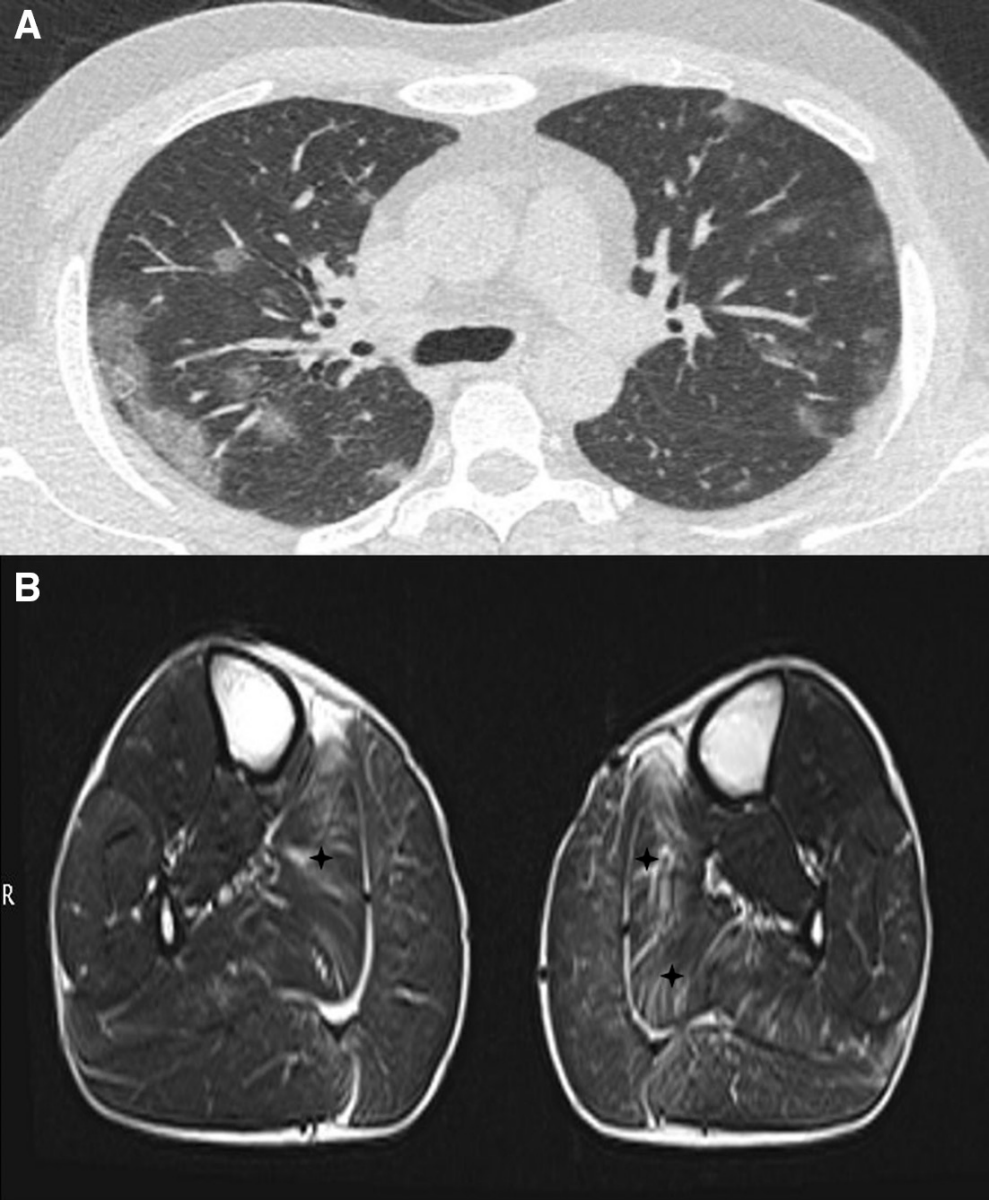
(A) CT scan showing ground-glass opacities and (B) MRI in T2 short-tau inversion recovery sequence showing bilateral gastrocnemius muscle oedema (asterisk).

On admission, blood work-up revealed creatine kinase (CK) of 19.250 IU/L (n<195 IU/L), C reactive protein (CRP) of 72 mg/L (n<5 mg/L), D-dimer of 1430 ng/ mL and lymphocytopenia. CT of the lung revealed bilateral ground-glass opacities (figure 1A). PCR testing for SARS-CoV-2 was positive. The results of influenza PCR were negative. A distal lower limb MRI showed bilateral gastrocnemius oedema, compatible with bilateral myositis (figure 1B). All immunological tests looking for any forms of myositis were negative. The diagnosis of COVID-19-associated myositis and pneumonitis was established, and a 5-day course of 1000 mg intravenous methylprednisolone, hydroxychloroquine and favipiravir was started. Over 5 days, his CK and CRP levels normalised. On day 10, a clear improvement in the patient’s general condition was observed, with an SpO_2_ of 97% without any need for supplemental oxygen.

Viral infections such as influenza A and B are well-known causes of myositis.^1^ A study performed in patients with COVID-19 reported that about 13.7% of these patients had elevated CK levels. Muscle weakness related to COVID-19 has been reported in two patients with the MRI documentation of such myositis.^2^  ^3^ We present an extremely rare case diagnosed with COVID-19-associated myositis.
